# Liquid Chromatography Mass Spectrometry (LC-MS) analysis in determining the saliva protein of orthodontic patients during retention phase

**DOI:** 10.4317/jced.55546

**Published:** 2019-03-01

**Authors:** Noor-Hidayah Awang-Kechik, Rohana Ahmad, Saeid-Reza Doustjalali, Negar-Shafiei Sabet, Aida-Nur-Ashikin Abd-Rahman

**Affiliations:** 1 Postgraduate, Centre for Paediatric Dentistry & Orthodontics Studies, Faculty of Dentistry, Universiti Teknologi MARA (UiTM), Malaysia; 2 Associate Professor, Faculty of Dentistry, Universiti Teknologi MARA, and Integrative Pharmacogenomics Institute, Puncak Alam Campus, Universiti Teknologi MARA, Malaysia; 3 Associate Professor, Faculty of Medicine, SEGi University, Kota Damansara, Selangor, Malaysia; 4 Deputy Dean, Centre for Paediatric Dentistry & Orthodontics Studies, Faculty of Dentistry, Universiti Teknologi MARA (UiTM), Malaysia

## Abstract

**Background:**

The biological responses involved during retention phase have been studied for many years but little is known about the effect of saliva proteome during retention phase of post-orthodontic treatment. This study aims to identify the protein profiles during retention phase in relation to biological processes involved by Liquid Chromatography Mass Spectrometry (LC-MS) approach.

**Material and Methods:**

A total of 5 ml of unstimulated saliva was collected from each subject (10 non-orthodontic patients and 15 post-orthodontic patients with 6-months retention phase). Samples were then subjected to LC-MS analysis. The expressed proteins were identified and compared between groups. Incisor irregularity for both maxilla and mandible were determined with Little’s Irregularity Index at 6-months retention phase.

**Results:**

146 proteins and 135 proteins were expressed in control and 6-months retention phase group respectively. 15 proteins were identified to be co-expressed between groups. Immune system process was only detected in 6-months retention phase group. Detected protein in immune system process was identified as Tyrosine-protein kinase Tec. Statistical significant of incisor irregularity was only found in mandible at 6-months retention phase.

**Conclusions:**

Our study suggests that immune system process protein which is Tyrosine-protein kinase Tec could be used as biomarker for prediction of stability during retention phase of post-orthodontic treatment.

** Key words:**Orthodontics, proteomics, retention, LC-MS, saliva.

## Introduction

Most orthodontic stability studies were carried out for long-term duration up to 20 years (1,2). Majority of the relapse findings were collected from the case records of patients such as dental casts. The length of retention, age at the start of treatment, angle classification, sex or any other dental cast or cephalometric measured variables were proven to be unable to serve as reliable predictors for future success of long-term stability. Relapse could be considered as any favourable change in tooth position away from a corrected malocclusion after orthodontic treatment (3). The degree of anterior irregularity that develops after retention is unpredictable and highly variable (3). The most commonly affected is mandibular labial segment compared to the upper labial segment (4). Other indicator to assess relapse are opening of the opening of the extraction site, rebound in increased overjet, overbite and change in inter-canine and inter-molar width (5).

Previously, orthodontic relapse more focused on the role of gingival and periodontal fibres (6). However, recent studies through molecular level have ruled out the other causes of relapse such as involvement of proteoglycans and glucosaminoglycans (7). With the advent of bio-molecular technology, attempts have been made to establish predictors of orthodontic relapse. Several biomarkers such as tartrate-resistant acid phosphatase (TRAP), alkaline phosphatase (ALP), osteonectin, collagen-1 and runx2 gen has been identified in relapse (8,9). Interestingly, similar biomarkers were also detected during active orthodontic tooth movement indicating that the same process occurs at two different situations (10).

Biomarker is an informative signal and could be used in diagnosing and predicting a specific condition. Biomarkers can be a cytokines, growth factors, hormone or any other factors that has a particular features and makes it instrumental for measuring disease progression or the effect of treatment (11). Effective biomarkers should be measureable in accessible body fluid such as serum, urine, blood, gingival crevicular fluid (GCF), saliva and also cerebrospinal fluid (CSF). However, saliva is a unique fluid and the interest in using it as a diagnostic medium has increased over the past decade due to several advantages over the other medium such as non-invasive, cost effective, easy to use, transport and store, stress-free collection and minimal risk of cross-infection (12).

LC-MS is one of the proteomic-based techniques that have been widely used in an understanding of a disease process, identification of novel diagnostic and prognostic biomarkers for human disease. It has become a suitable method due to its relative convenience, high sensitivity and specificity and high in throughput potential (13).

In this study, we aimed to determine differentially expressed salivary proteins between non-orthodontic patient and 6-months retention phase of orthodontic patients with the intention that the detected biomarkers could be used for prediction of stability post-orthodontic treatment.

## Material and Methods

- Ethics statement

This is a prospective cohort clinical study with convenience sampling. This study was carried out with approval from Research and Ethics committee, Universiti Teknologi MARA (600-IRMI 5/1/6)/2016). Adult subjects and parents or guardians were given an information sheet and informed consent was obtained before participating in the study.

- Patient selection

All included patients were drawn from patients nearly at the debond stage. They had undergone orthodontic treatment with four premolars extraction with MBT prescription 0.022 x0.028-in slot pre-adjusted edgewise-fixed orthodontic appliances (Victory SeriesTM, 3M Unitek, Germany) and followed by 6-months retention phase. Patients were given Hawley retainers and instructed to wear it for night-time only (14). Enrollment started in December 2016 until October 2017. All patients were generally systemically healthy and presented with good periodontal status. Patients with poor oral hygiene, smoker, pregnant and on bonded retainers were excluded. Ten healthy non-orthodontic patients were recruited as the control group.

- Incisor irregularity measurement

Incisor irregularities at debond and 6-months retention phase was measured using Little’s irregularity Index (15). The digital dial caliper was used to measure the study model to an accuracy of 0.01 mm (Germany Stainless HSL 246-15) (2). Study model of other 15 patients were randomly selected for calibration purpose in two separate occasions 2 weeks apart. Intra class correlation coefficient (ICC) was used.

- Saliva collections and processing

Saliva samples were collected from each patient at 6-months retention phase. Non-traumatic procedures were carried out to prevent any bleeding. Patients were seated in an upright position and were asked to rinse their mouth first with distilled water and then to rest for 5 minutes before saliva collection. A total of 5 ml of unstimulated whole saliva were collected by drooling into 50 ml sterile plastic centrifuge tube (16). Patients were asked to avoid doing any oral activity, i.e no food consumption 2 hours prior to collection and no liquid consumption 30 minutes prior to saliva collection. During the collection process, patients were asked not to speak or move their tongue. The head was tilted downwards to facilitate the secreted saliva to accumulate in the mouth. Saliva samples were collected for 7 minutes. After collection, the saliva samples were kept on ice and then immediately centrifuged at 10,000 rpm, 40 C for 10 minutes to remove insoluble materials, cells and debris (17). The supernatants were collected and pellets were discarded. Each sample was stored at -80 °C until further analysis. Saliva protein concentration was determined using Bradford assay (Bio-Rad, Hercules, CA, USA) (18). Moreover, throughout the study, all subject received repeated oral hygiene instruction and education to maintain their oral hygiene. The same procedures of saliva collection were also subjected to control group.

- Liquid Chromatography Mass Spectrometry (LC-MS)

Approximately 100 µg of proteins from each sample was mixed with 100 µL of 6 M urea in 50 mM Tris-HCl, pH 8.0. Next, 5 µL of 200 mM dithitreitol (DTT) in 50 mM ammonium bicarbonate, pH 8.0 was added into the mixture and incubated at room temperature for an hour. Then, 20 µL of 200 mM Iodoacetamide (IAA) in 50 mM ammonium bicarbonate, pH 8.0 was added into the mixture followed by incubation at room temperature in the dark. The excess IAA was chelated by the addition of 20 µL of 50 mM DTT in 50 mM ammonium bicarbonate, pH 8.0 into the mixture which was then incubated in the dark for another 1 hour at room temperature. 775 µL of 50 mM ammonium bicarbonate was added into the mixture to reduce the concentration of urea to 0.6 M prior to the addition of trypsin. Then, 2 µg MS grade trypsin (Thermo, USA) was added into the mixture giving final ratio of 1: 50 (w/w, trypsin:protein).

The mixture was vortexed gently and incubated at 37°C for at least 18 hours. Finally, 2 µL of neat formic acid was added to digested protein mixture to stop trypsin activity before it was stored at -20°C until further use. At the end of the tryptic digestion, the protein complexes of the whole cell lysates were reduced into peptide complexes. The peptide complexes were desalted using C18 spin column (Thermo, USA) following equilibration in 50% acetonitrile (ACN) in 0.1% formic acid. The desalted peptides were then eluted and flown through a 5-95% gradient of acetonitrile (ACN) through an LC system (Agilent 1200 Series HPLC-Chip/MS, Agilent, USA) into a HPLC chip configuration consisting of a 160 nL enrichment column and a 150 mm x 75 µm analytical column (Zorbax 300SB-C18). The mobile phases used were: A) 0.1% formic acid in water and B) 90% acetonitrile with 0.1% formic acid. A 60 minutes long gradient method was used for the LC separation.

In this study, the software PeptideShaker was used as the search engine where it employs the X!Tandem search algorithm (19). PeptideShaker was configured to search the MS/MS spectra acquired from the nanoLC-ESI-QTOF MS/MS that match the ions profiled generated from the whole proteome sequences of human retrieved from the Uniprot database. The whole proteome sequence was provided in form of a multi-FASTA file and crucial parameters like fixed modification, variable modification and proteolytic enzyme were set to carbamidomethylation of cysteine, oxidation of methionine and trypsin respectively. The rest of the parameters were left as default.

- Comparison of protein profiles between groups 

The differential expressions of proteins were detected by comparing the data between groups. A Venn diagram was used (bioinformatics.psb.ugent.be). Further details of biological processes involved were determined by using PANTHER (Protein Analysis Through Evolutionary Relationship) Classification System version 13.1 (http://www.pantherdb.org). Each group of proteins were classified according to it biological process and displayed in the form of the percentage (20).

- Statistical analysis

Clinical data collected were analyzed using Statistical Package for the Social Science (SPSS) version 21.0 (SPSS® Inc., Chicago, IL USA). Paired t-test were used to assess the difference of irregularity index between at debond and 6-months retention phase.

## Results

- Demographic data

A total number of 25 patients were recruited including 10 patients as control. The samples consisted 18 female (72%) and 7 males (28%). The average age for control group was 24.9 years (SD 4.91) and 21 years (SD 3.68) for 6-months retention group.

- Measurement of irregularity index 

The agreement score between 2 operators using ICC is 0.99 with 95% confidence interval. Intra-operator reliability also showed an excellent agreement with ICC is 0.99. There was statistical significant difference between mandibular irregularities from debond to 6-months retention phase ( Table 1).

**Table 1:**
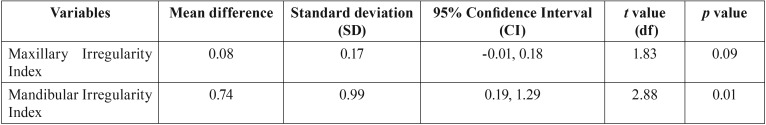
Paired t-test of maxillary and mandibular irregularity index.

- Total number of proteins detected in control and 6-months retention phase group

A total number of 281 proteins were identified. 146 and 135 proteins were found in control group and 6-months retention phase group.

- Comparison of proteins detected between control and 6-months retention phase group

15 types of proteins were identified to be co-expressed between control and 6-months retention phase group. Further details of detected proteins are listed in  Table 2.

**Table 2:**
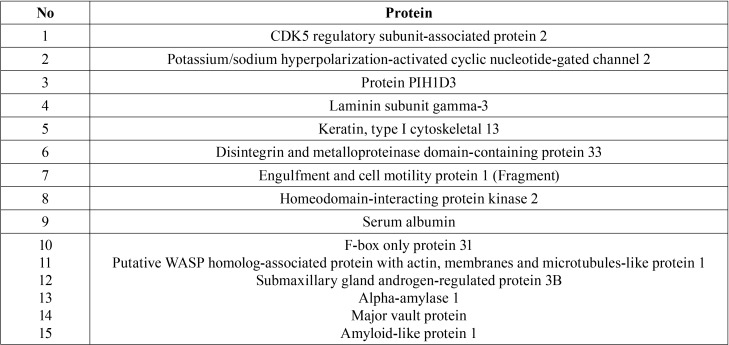
Co-expressed proteins in control and 6-months retention phase group.

- Identification of biological processes

Bioinformatics analysis of proteome using PANTHER database has identified eleven biological processes that are related to the protein expressed in all groups. Percentage of proteins involved in each biological process is listed in  Table 3.

**Table 3:**
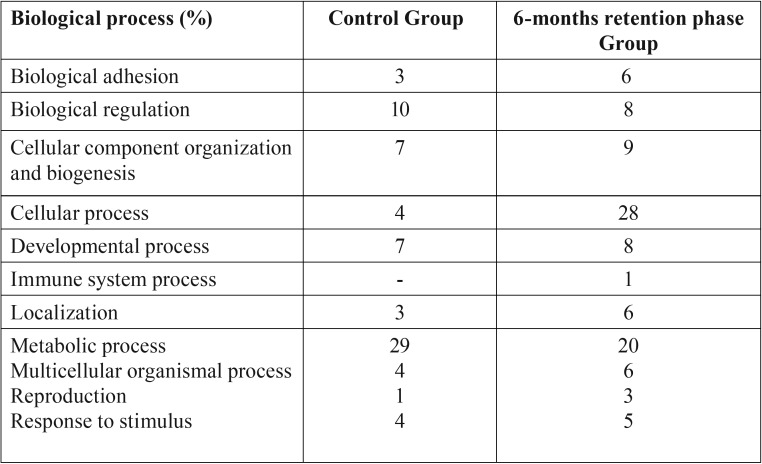
Percentage of identified proteins in relation to biological process.

## Discussion

There were more female patients compared to male. Females are more likely to have a greater desire to seek orthodontic treatment. Furthermore, females are more concern about their dental appearance than males (21).

Results collected from this study also revealed that the incisor irregularity is most marked in the mandibular labial segment compared to the upper labial segment. Incisor irregularity is considered to be the most noticeable features to the orthodontists and patients. Moreover, relapse of the anterior teeth alone gives high weightage in any assessment of the stability of treatment (22).

In this study, on the first day after debonding, all patients were advised to wear their Hawley retainers only at night-time basis. This regime was chosen as it has been reported by Shawesh *et al.* (2010), there is an equal post-orthodontic treatment stability results following either full or part-time wear of Hawley retainers during retention phase (14).

To date, there have been no studies investigating the protein profiles during retention phase using salivary proteomics. Previous studies more concentrated on searching of biomarkers during active orthodontic tooth movement (23). Various groups of biomarkers such as pro and anti-inflammatory, enzymes, bone deposition and resorption biomarkers have been recognized and could be used in monitoring the progress of active orthodontic tooth movement. Several studies through histomorphometric and gingival crevicular fluid (GCF) found that increased in alkaline phosphatase (ALP) and tartrate resistance acid phosphatase (TRAP) during relapse period (24,25). These biomarkers also could be used in monitoring the stability of post-orthodontic treatment during retention phase.

In this recent study, unique proteome from the human saliva that have undergone orthodontic treatment have been identified. Through qualitative analysis of LCM-MS, 146 proteins in control group and 135 proteins in 6-months retention phase group were detected. The LC-MS technique used in this study is sensitive in recognizing large mass range of peptides. Other study used different proteomics technique such as matrix-assisted laser desorption/ionization time-of-flight mass spectrometry (MALDI-TOF MS) and found an average 144 protein mass peaks (26). Through surface-enhanced laser desorption/ ionization time-of- flight mass spectrometry (SELDI-TOF MS), four different proteins at m/z 3372, 5232, 4045 and 10128 were significantly higher after three months of active orthodontic treatment (27). Thus, mentioned studies support that salivary proteomics methods could be used to analyze peptide profiles either during active orthodontic treatment or during retention phase of post-orthodontic treatment. This method is then useful in studying relapse during retention phase as remodeling of periodontal tissues occurred during active and also post-orthodontic treatment.

Further analysis of detected proteins through PANTHER database has identified unique proteins expressed in control and 6-months retention phase group. Almost 50% of proteins belong to cellular and metabolic process. Both of these processes are important for ensuring continuous growth of healthy cells. The other remaining proteins are belonging to other various biological processes as shown in  Table 3.

Meanwhile, 15 proteins were identified to be co-expressed in both control and 6-months retention group. All of the identified proteins are involved in cell binding and receptor activity that govern the cellular parts, extracellular matrix and extracellular region of the cell (20). Furthermore, most of the identified proteins regulate cell growth among other functions.

Further details of analysis on biological process revealed that, there was one percent of protein is related to the immune system during 6-months retention phase. However, there were no proteins related to the immune system was detected in the control group. Protein that was identified to have roles in the immune system is known as Tyrosine-protein kinase Tec (Tec protein). Tec has been recognised as a factor that directly involves in unconventional secretion of Fibroblast Growth Factor (FGF) (28), and play role in regulating the activation and development of several cells such as T cell, B cell and mast cells (29). Moreover, Tec kinase also has been proven to be involved in receptor activator of nuclear kappa B ligand (RANKL)-induced osteoclastogenesis (30). Meanwhile, post-orthodontic treatment with relapse also detects the involvement of RANKL (8). Thus, it can be suggested that, by the presence of Tec kinase at 6-months retention phase, it might indicate that bone remodeling still occur.

It has to be noted that this study consisted imbalance in sample size population. We were able to collect only 15 patients with involvement of more females compared to male. Furthermore, expressed proteins could potentially serve as biomarkers for post-orthodontic treatment stability. However, the cost of the analyses and the equipment needed may limit the use of proteomics as diagnostic tool. The results obtained in this study were qualitative. Further validation is needed to obtain better and concise results.

To the best of our knowledge, this study represents the first attempt of using LC-MS to identify expression of saliva proteins in retention phase post-orthodontic treatment. Our study suggests that immune system process protein which is Tyrosine-protein kinase Tec could be used as biomarkers for prediction of stability post-orthodontic treatment.
